# Concurrent frontal and parietal network TMS for modulating attention

**DOI:** 10.1016/j.isci.2022.103962

**Published:** 2022-02-22

**Authors:** Stefano Gallotto, Teresa Schuhmann, Felix Duecker, Marij Middag-van Spanje, Tom A. de Graaf, Alexander T. Sack

**Affiliations:** 1 Section Brain Stimulation and Cognition, Department of Cognitive Neuroscience, Faculty of Psychology and Neuroscience, Maastricht University, 6229 Maastricht, the Netherlands; 2 Maastricht Brain Imaging Centre, 6229 Maastricht, the Netherlands; 3 Centre for Integrative Neuroscience, Maastricht University, 6200 Maastricht, the Netherlands; 4 Department of Psychiatry and Neuropsychology, School for Mental Health and Neuroscience (MHeNs), Brain+Nerve Centre, Maastricht University Medical Centre+ (MUMC+), 6224 Maastricht, the Netherlands; 5 InteraktContour, 8070 Nunspeet, the Netherlands

**Keywords:** Neuroscience, Systems neuroscience, Techniques in neuroscience

## Abstract

Transcranial magnetic stimulation (TMS) has been applied to frontal eye field (FEF) and intraparietal sulcus (IPS) in isolation, to study their role in attention. However, these nodes closely interact in a “dorsal attention network”. Here, we compared effects of inhibitory TMS applied to individually fMRI-localized FEF or IPS (single-node TMS), to effects of simultaneously inhibiting both regions (“network TMS”), and sham. We assessed attention performance using the lateralized attention network test, which captures multiple facets of attention: spatial orienting, alerting, and executive control. TMS showed no effects on alerting and executive control. For spatial orienting, only network TMS showed a reduction of the orienting effect in the right hemifield compared to the left hemifield, irrespective of the order of TMS application (IPS→FEF or FEF→IPS). Network TMS might prevent compensatory mechanisms within a brain network, which is promising for both research and clinical applications to achieve superior neuromodulation effects

## Highlights

• Compensation happens within a given brain network after disrupting one of its nodes
• Simultaneous FEF-IPS TMS leads to stronger effects compared to single-node TMS
• Network TMS might overcome brain network compensation within the DAN
• Network TMS might open new avenues of experimentation and treatment

## Graphical abstract

**Figure:**
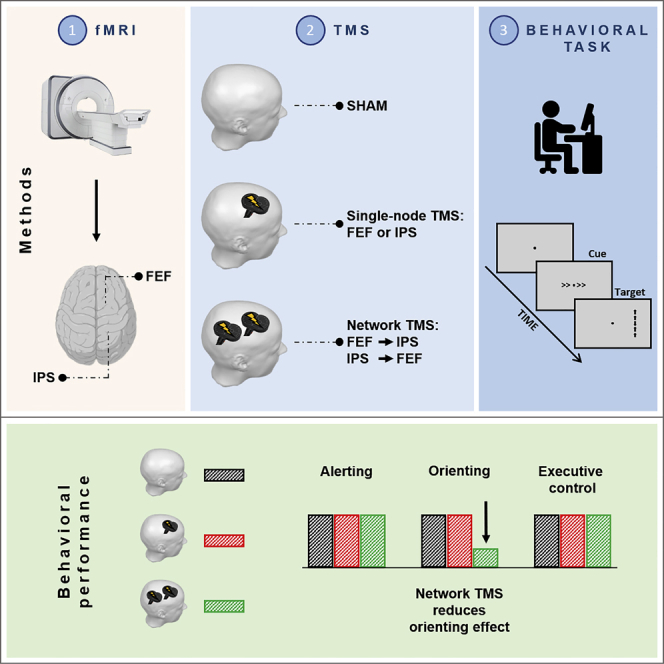

## Introduction

Cognition is accomplished by the interaction of several regions in the brain which together form networks (Cocchi et al., 2013; Sporns, 2013). The voluntary control of visuospatial attention is supported by the dorsal attention network (DAN), consisting primarily of the frontal eye field (FEF) in frontal cortex and the intraparietal sulcus (IPS) in parietal cortex (Corbetta and Shulman, 2002; Mayrhofer et al., 2019). Evidence has shown that these regions interactively modulate activity in visual cortices to achieve the preferential processing of (stimuli in) a particular part of our visual field (Moore and Fallah, 2004; Noudoost et al., 2010). In healthy participants, brain imaging using fMRI reveals activity in this network during attention shifts (Corbetta et al., 2000; Hopfinger et al., 2000; Kastner et al., 1999), with its connectivity being a predictor of performance in attention tasks (Szczepanski et al., 2013). The relevance of the DAN for attention processes has also been extensively confirmed in clinical populations, showing that lesions to either FEF or IPS impair performance on attention-orienting tasks (Bartolomeo et al., 2012; Corbetta et al., 2005; Duncan et al., 1999; Karnath et al., 2002; Mesulam, 1999) and often lead to hemispatial neglect (Corbetta et al., 2005). This syndrome is more commonly observed after impairment of the right hemisphere, thus suggesting right hemispheric dominance (Duecker et al., 2013a; Mayrhofer et al., 2019; Mesulam, 1981; Sack, 2010).

Transcranial magnetic stimulation (TMS) has been repeatedly applied to establish the causal contribution of single nodes forming networks (Duecker et al., 2013a; Ruff et al., 2009; Sack et al., 2002, 2007; Sack et al., 2005; Thut et al., 2005) as well as the functionality of the networks themselves (Bortoletto et al., 2015; Eldaief et al., 2011; Hampson, 2010; Sack et al., 2005, 2007). Concerning the DAN, inhibitory TMS protocols to either the left or right FEF were shown to affect endogenous attention performance (Duecker et al., 2013b; Grosbras and Paus, 2002; Marshall et al., 2015). Similarly, TMS to parietal cortex has been reported to modulate performance on visuospatial tasks (Sack et al., 2002), including attention orienting (Thut et al., 2005) and extinction (Battelli et al., 2008; Dambeck et al., 2006; Hilgetag et al., 2001).

Given the interactive nature of brain areas and brain networks (Cocchi et al., 2013; Lee et al., 2019), the effects of TMS are not limited to the target regions but do spread to other short- and long-distance areas of the network they are part of (Bestmann et al., 2008; Eldaief et al., 2011; Feredoes et al., 2011; Ilmoniemi et al., 1997; Morishima et al., 2009; Ruff et al., 2008; Sack et al., 2007; Werf, Sanz-arigita, Menning and Heuvel, 2010) as well as to other overlapping networks (Chen et al., 2013; Gratton et al., 2013; Lee et al., 2019). This, in turn, makes it often difficult to draw strong conclusions about which node of a given network plays which role. In fact, different mechanisms might take place within the network to counteract the effects of disruption and rebalance its activity to the baseline level (Hartwigsen, 2018). For example, behavioral consequences of a single-node TMS disruption may be unmasked only when also blocking the compensatory response of a second node using a sequential TMS disruption approach (Sack et al., 2005). Therefore, it would be crucial to test whether stimulating multiple (interactive) nodes of a network affects behavior differently from the more conventional single-node TMS. This approach would allow a more comprehensive understanding of the mechanisms employed by cognitive networks in response to local insults.

To address this question, the current study used continuous theta burst stimulation (cTBS) to reduce cortical excitability (Huang et al., 2005) in the DAN, targeting FEF and IPS either in isolation, or concurrently, with sequential cTBS interventions. We targeted the DAN nodes and therefore expected to observe behavioral changes mainly on attention-orienting performances (Duecker et al., 2013a; Grosbras and Paus, 2002; Marshall et al., 2015; Thut et al., 2005). Considering that the DAN interacts also with other attention networks (Callejas et al., 2005; Callejas et al., 2004; Chica et al., 2012; Petersen and Posner, 2012; Posner and Petersen, 1990), we used a task able to capture not only orienting mechanisms but also other facets of attention to more completely assess the consequences of our neuromodulation procedures. The lateralized attention network test (LANT) was explicitly designed with the purpose of behaviorally quantifying spatial orienting, executive control, and alerting (Asanowicz et al., 2012; Callejas et al., 2004, 2005; Chica et al., 2012; Greene et al., 2008). Thus, the use of this task allowed us to assess orienting mechanisms but also alerting and executive control mechanisms, which are partly subserved by areas overlapping with the DAN as well as other networks, allowing the assessment of potential TMS modulations also across different networks interacting with the DAN.

Because cTBS should impair cortical excitability for up to an hour (Huang et al., 2005), and cTBS was applied to both sites in immediate succession, we consider this a simultaneous, concurrent, network inhibition protocol. Thus, administration was sequential, but effects on task simultaneous. However, this is based on the hypothesis that the order of sequential stimulations (IPS→FEF or FEF→IPS) is irrelevant for this task. The inclusion of both orders as separate experimental conditions allowed confirmation of this hypothesis, immediately allowing an internal replication of any network TMS effects on behavior. Indeed, looking ahead, we did not find significant effects of stimulation on spatial orienting after IPS or FEF inhibition in isolation, but we found spatial orienting effects after disruption of both regions, independently of the order of stimulation. Neither single nor double stimulation modulated reaction times of alerting and executive control. As discussed more extensively below, the modulation observed on orienting after network TMS supports the hypothesis that simultaneous inhibition of two nodes of the same network can have more robust (superadditive) modulatory effects on a task thought to be subserved by such a network, as compared to conventional single-node stimulation. Because many cognitive functions have a neuronal network basis, this approach has important implications, and offers a relatively straightforward new avenue of experimentation and treatment in both research and clinical contexts.

## Results

### LANT attention components validation

**Figure 1:**
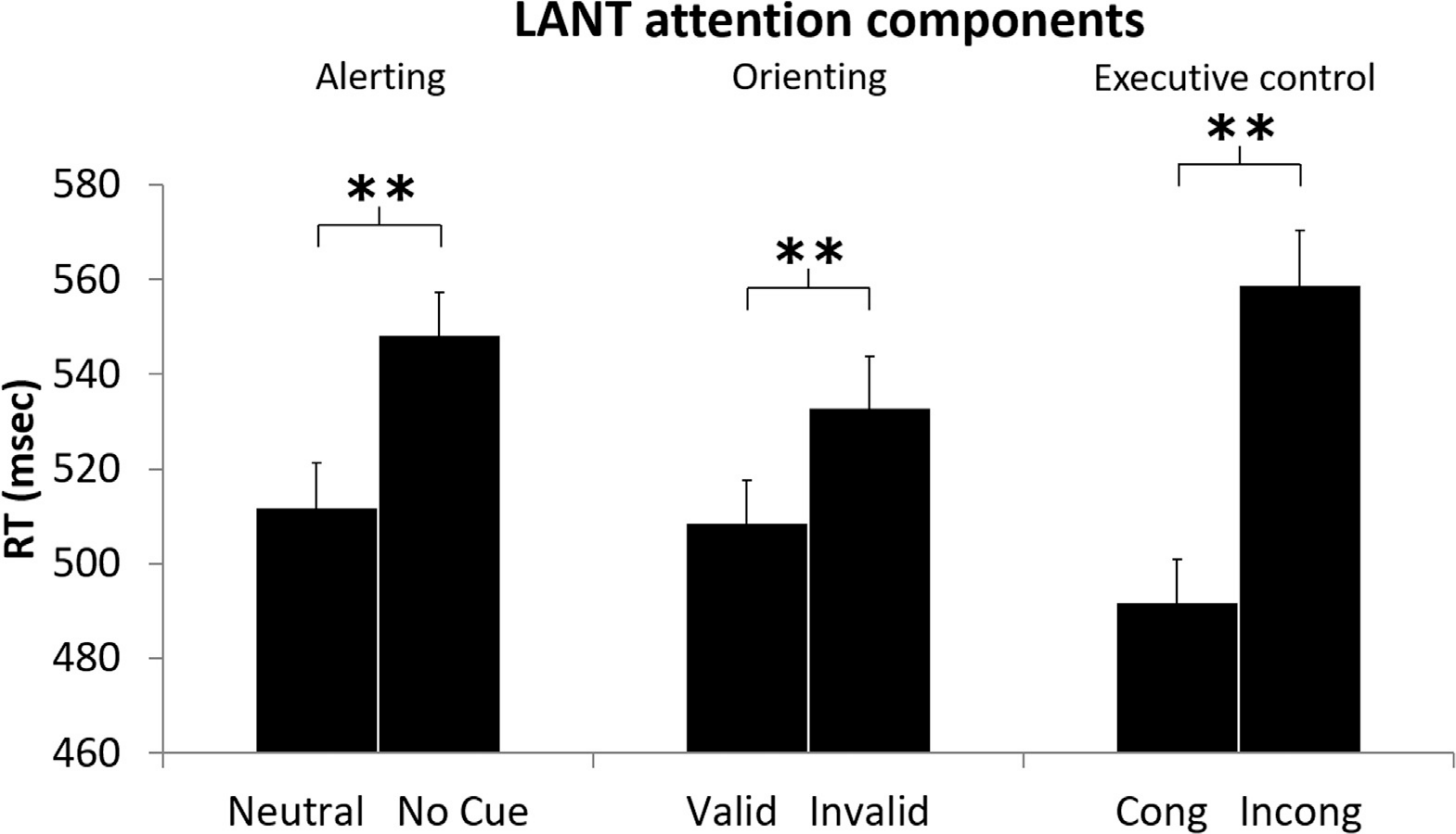
LANT attention components validation RT of cue and target conditions used to obtain the three main attention components: alerting (RT difference between neutral and no cue trials), spatial orienting (RT difference between valid and invalid cue trials), and executive control (RT difference between congruent and incongruent trials) in the sham condition. Error bars depict standard error of the mean (SEM). Two asterisks represent a significant difference between bars (p < 0.01).

First, we evaluated whether we could replicate previously reported effects of cues/targets on reaction times in our implementation of the LANT, based on the data from the sham condition (without TMS modulation). Targets preceded by a neutral cue were identified faster than targets presented without any cue (*t*_(19)_ = −10.116, p < 0.001, *d* = −2.262), showing an alerting effect. Validly cued targets were identified faster than invalidly cued targets (*t*_(19)_ = −4.896, p < 0.001, *d* = −1.095), showing a spatial orienting effect. Congruent targets were identified faster than incongruent targets (*t*_(19)_ = −7.748, p < 0.001, *d* = −1.732), showing an executive control effect. Results are reported in Figure 1. Having established the expected LANT effects reflecting alerting, spatial orienting, and executive control, we then evaluated for each of these effects whether and how TMS to IPS, FEF, or both, modulated them.

### Overall reaction times

Table 1 reports raw reaction times for congruent and incongruent trials, the four types of cue, and the two hemifields in the five TMS conditions. Below, we report effects of TMS for the three attention components captured by the LANT.

**Table 1:** Reaction times of all LANT conditions

Condition		CVL	CVR	CNL	CNR	CIL	CIR	CNoL	CNoR	IVL	IVR	INL	INR	IIL	IIR	INoL	INoR
SHAM	Av	478	471	485	474	501	496	522	509	544	542	550	539	567	568	584	578
	SE	9.0	8.9	9.0	9.8	12.0	11.5	8.6	8.9	9.4	13.5	10.9	12.3	12.8	13.6	11.9	14.2
FEF	Av	478	469	488	471	503	492	528	521	545	538	556	546	572	573	588	571
	SE	9.0	8.2	10.0	8.3	9.8	8.9	11.0	11.2	11.3	12.2	11.8	14.2	14.0	13.8	12.9	12.4
IPS - > FEF	Av	476	470	484	472	502	485	528	512	550	541	558	543	571	556	591	571
	SE	9.6	9.7	9.0	9.5	10.1	9.1	11.6	12.1	12.2	14.6	11.7	14.7	12.9	11.5	13.5	14.0
IPS	Av	473	459	481	461	503	482	519	505	543	534	554	537	563	556	589	572
	SE	9.0	8.2	8.4	7.4	11.1	9.4	10.9	9.8	11.4	13.4	12.0	14.4	13.5	16.0	14.0	13.5
FEF - > IPS	Av	478	463	487	465	510	495	525	510	546	541	561	537	584	556	596	572
	SE	7.4	7.7	7.9	7.9	9.7	9.4	9.2	8.6	10.1	14.1	11.1	13.5	11.4	14.1	11.4	11.9

1^st^ letter: C = congruent, I = incongruent; 2^nd^ letter: V = Valid, N = Neutral, I = invalid, No = No cue; 3^rd^ letter: L = left, R = right; Av = average; SE = standard error.

### No TMS effects on alerting

**Figure 2:**
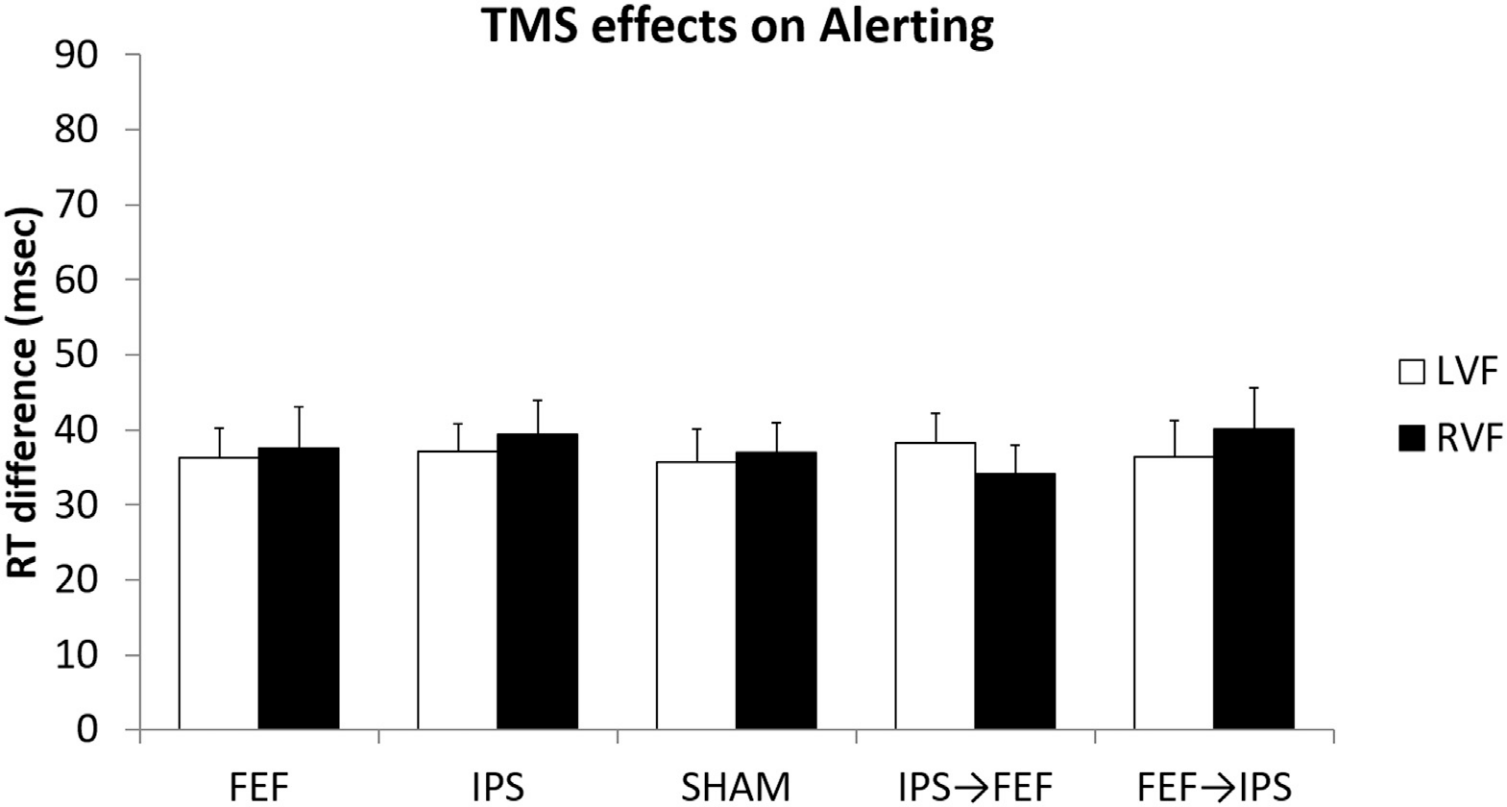
No TMS effects on alerting RT differences between not cued target locations (no cue trials) and temporally cued target locations (neutral trials) obtained from the 5 TMS conditions (single-node TMS: FEF = Frontal Eye Field, IPS = Intraparietal Sulcus, sham, and network TMS: IPS→FEF, FEF→ IPS). Error bars depict SEM.

A repeated-measures ANOVA performed on the alerting effect (difference in reaction time between neutral and no cue trials) showed no main effect of Hemifield (*F*_(1,19)_ = 0.143, p = 0.709, *ƞ*^*2*^ = 0.000), no main effect of TMS (*F*_(4,76)_ = 0.089, p = 0.986, *ƞ*^*2*^ = 0.002), and no TMS × Hemifield interaction (*F*_(4,76)_ = 0.550, p = 0.700, *ƞ*^*2*^ = 0.005). Thus, mean reaction times of the alerting effect after active stimulation over right FEF and/or IPS did not differ from the mean reaction times of the alerting effect in the sham condition in the left nor the right hemifield. Results are reported in Figure 2.

### Network TMS effects on spatial orienting

**Figure 3:**
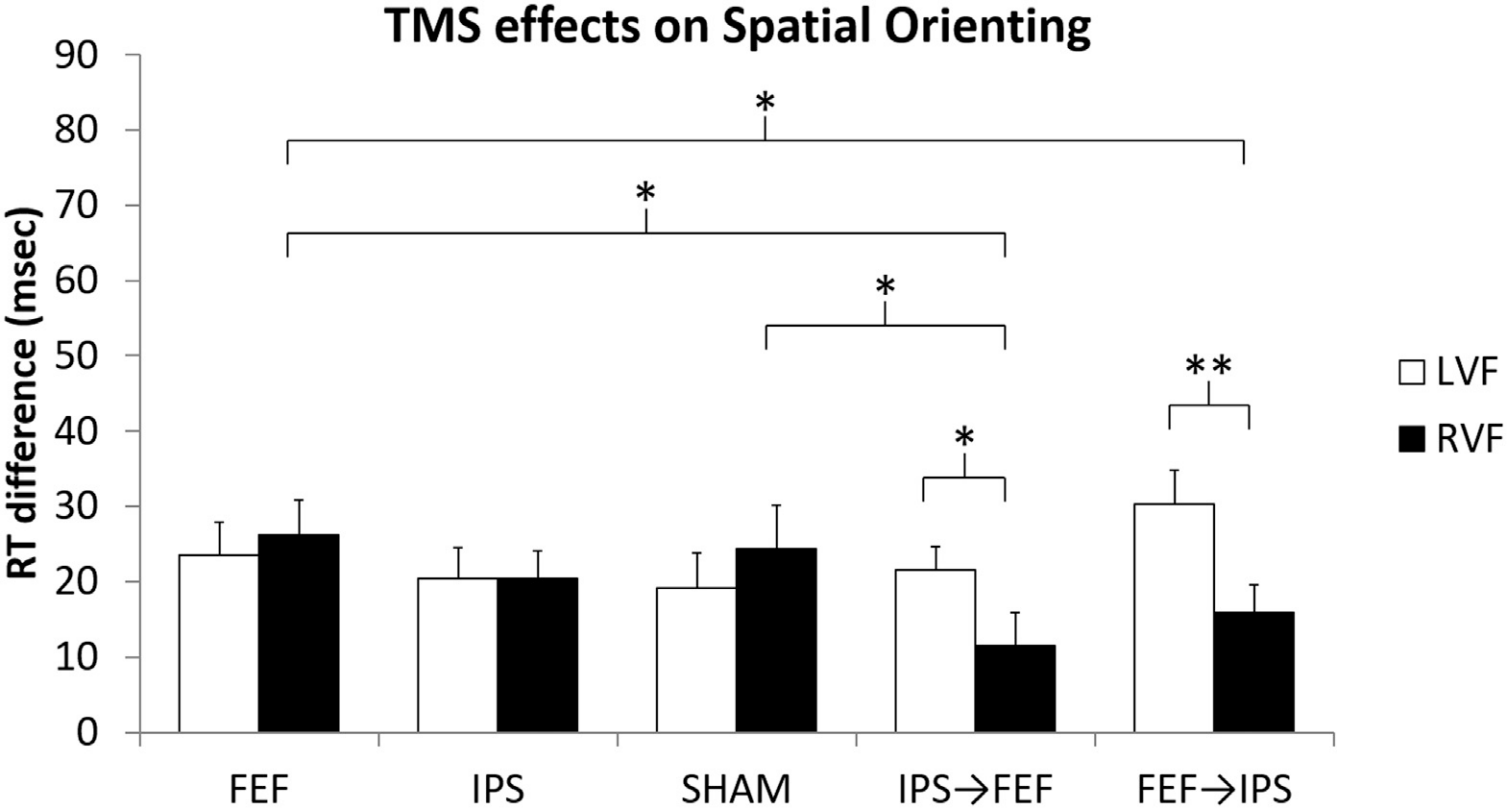
Network TMS effects on spatial orienting RT differences between validly cued target locations (valid trials) and invalidly cued target locations (invalid trials) obtained from the five TMS conditions (single-node TMS: FEF = Frontal Eye Field, IPS = Intraparietal Sulcus, sham, and network TMS: IPS→FEF, FEF→IPS). Error bars depict SEM One and two asterisks represent a significant difference between bars (p < 0.05 and p < 0.01 respectively).

A repeated-measures ANOVA performed on the orienting effect (difference in reaction time between valid and invalid cue trials) showed no main effect of Hemifield (*F*_(1,18)_ = 3.738, p = 0.069, *ƞ*^*2*^ = 0.007), no main effect of TMS (*F*_(4,72)_ = 1.138, p = 0.345, *ƞ*^*2*^ = 0.022), and a significant TMS × Hemifield interaction (*F*_(4,72)_ = 3.514, p = 0.011, *ƞ*^*2*^ = 0.039). A further exploration of the interaction using follow-up t-tests showed that the orienting effect after active stimulation of both network nodes was much more attenuated in the right as compared to the left visual field independently of the order of stimulation (IPS→FEF: *t*_(18)_ = 2.140, p = 0.046, *d* = 0.491; FEF→IPS: *t*_(18)_ = 3.918, p = 0.001, *d* = 0.899). To better isolate whether this effect was coming from the left or the right hemifield, we also tested differences within the same hemifield across TMS conditions. In the right hemifield, the orienting effect was attenuated after active stimulation of both nodes compared to single-node stimulation of the FEF (IPS→FEF: *t*_(18)_ = 2.836, p = 0.011, *d* = 0.651; FEF→IPS: *t*_(18)_ = 2.141, p = 0.046, *d* = 0.491), and compared to sham after active stimulation of both nodes when IPS was stimulated first (IPS→FEF: *t*_(18)_ = 2.246, p = 0.038, *d* = 0.515). This was not the case when IPS was the second node being stimulated (FEF→IPS: *t*_(18)_ = 1.142, p = 0.268, *d* = 0.262). No differences were observed between network TMS and single-node stimulation of the IPS (IPS→FEF: *t*_(18)_ = 1.678, p = 0.111, *d* = 0.385; FEF→IPS: *t*_(18)_ = 1.311, p = 0.206, *d* = 0.301). In the left hemifield, no differences between network TMS, single-node stimulation of the FEF or IPS, or sham were observed. Results are reported in Figure 3.

### No TMS effects on executive control

**Figure 4:**
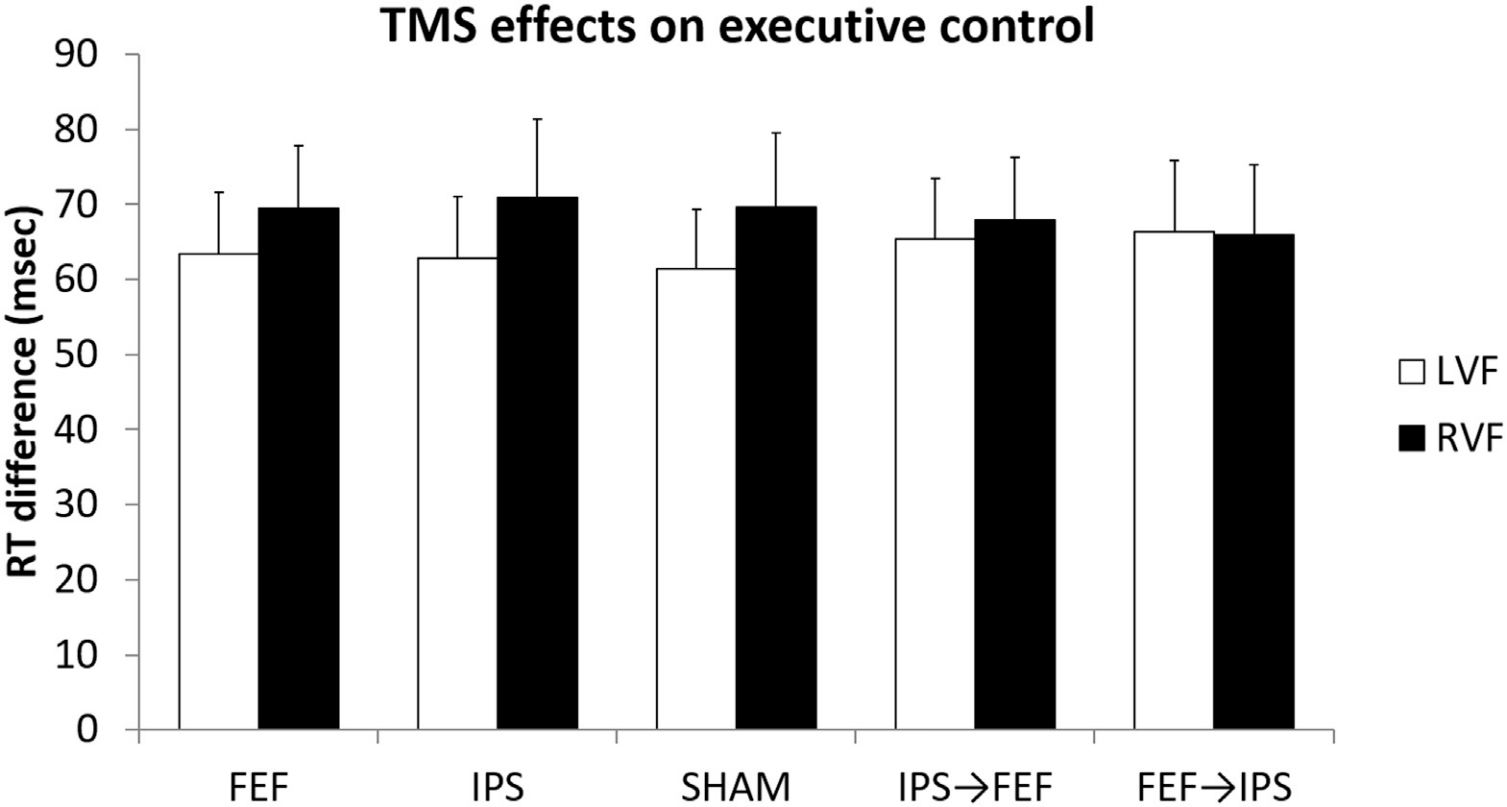
No TMS effects on executive control RT differences between congruent trials and incongruent trials obtained from the five TMS conditions (single-node TMS: FEF = Frontal Eye Field, IPS = Intraparietal Sulcus, sham, and network TMS: IPS→FEF, FEF→IPS). Error bars depict SEM.

A repeated-measures ANOVA performed on the executive control effect (difference in reaction time between congruent and incongruent trials) showed no main effect of Hemifield (*F*_(1,19)_ = 0.081, p = 0.780, *ƞ*^*2*^ = 0.000), no main effect of TMS (*F*_(4,76)_ = 0.427, p = 0.789, *ƞ*^*2*^ = 0.001), and no TMS × Hemifield interaction (*F*_(4,76)_ = 1.545, p = 0.198, *ƞ*^*2*^ = 0.002). Thus, mean reaction times of the executive control effect after active stimulation over right FEF and/or IPS did not differ from the mean reaction times of the executive control effect in the sham condition in the left nor the right hemifield. Results are reported in Figure 4.

## Discussion

The present study aimed at inhibiting two nodes of the right DAN in isolation, and concurrently, with the explicit goal to achieve additive, superadditive, or more consistent behavioral effects after network TMS as compared to single-node TMS. To assess behavioral performance, we used the Lateralized Attention Network Test (LANT, Asanowicz et al., 2012; Callejas et al., 2004, 2005; Chica et al., 2012; Greene et al., 2008; Posner, 1994) which inherently offers the possibility to assess alerting, spatial orienting, and executive control mechanisms. cTBS to FEF or IPS in isolation did not significantly affect spatial orienting, whereas cTBS to both DAN nodes (IPS→FEF and FEF→IPS conditions) had a significant effect.

### Network (but not single-node) TMS effects on spatial orienting

In contrast to previous studies (for an in-depth review see Mengotti et al., 2020), we did not find significant effects on spatial orienting after TMS-induced inhibition of FEF or IPS in isolation. Modulation of spatial orienting performance only occured after simultaneous inhibition of both nodes, independently of the order of stimulation (IPS→FEF or FEF→IPS). A variety of factors might have contributed to these results.

First, the efficacy of a TMS protocol depends on several variables such as the distance of the target region from the skull (Stokes et al., 2007), the intensity used, the intrinsic connectivity level and physiological state of the network this region is part of (Hawco et al., 2018; Sack et al., 2007; de Graaf et al., 2009), and the TMS protocol that is applied (Nahas et al., 2001; Nettekoven et al., 2015). We used a cTBS protocol with rather low stimulation intensities (80% rMT). It may thus be possible that in some participants the efficacy of stimulation was not sufficiently high to induce the desired behavioral changes when stimulating a single node of the network. Second, compensation mechanisms after brain stimulation have been demonstrated within the targeted region (Lee et al., 2003; O’Shea et al., 2007), in connected regions within the same network (Hartwigsen et al., 2013; Jung and Lambon Ralph, 2016; Sack et al., 2005), and even in other networks (Noppeney et al., 2004; Price and Friston, 2002). It may therefore also be speculated that single disruption of FEF and IPS did not lead to any significant behavioral effects due to compensatory processes within other nodes of the network that allowed maintaining performance despite the local perturbation. This concept of obscured or masked behavioral effects of single-node TMS has been demonstrated before in the parietal cortices (Sack et al., 2005), showing that the (lack of) behavioral effects in TMS studies not only may reveal the (absence of) functional relevance of a given brain region but also often in fact shows how the rest of the brain copes with this local brain insult (Sack et al., 2005; de Graaf and Sack, 2011). The funtional-anatomical models of attention control clearly describe specific fronto-parietal interactions within and across the DAN that, in principle, could allow several brain mechanisms to counteract the effects of single-node TMS in order to rebalance the activity of the network to its baseline level (Hartwigsen, 2018).

Finally, the task itself, the LANT, may be a factor. It is in fact known that different tasks are differentially susceptible to TMS modulation (Romei et al., 2016). Previous TMS studies used (modified) Posner (Capotosto et al., 2012; Duecker et al., 2013a; Marshall et al., 2015), line bisection (Fierro et al., 2000; Pourtois et al., 2001; Ricci et al., 2012; Salatino et al., 2014), or visual extinction (Battelli et al., 2008) paradigms. However, only a few recent studies showed modulation of attention performances by combining repetitive TMS protocols applied to the DAN with the attention network test (Xu et al., 2013, 2016). Here, we even used a more specific version of the ANT (i.e. the LANT), asking participants to covertly shift their attention laterally. Because this process vastly engages the DAN, it might then be that different stimulation parameters are needed to achieve the same effects than what is usually employed.

### Directions of effects

Although we expected (and found) the strongest effects in the network TMS conditions, and specifically on the spatial orienting component of the task, it could be considered surprising that inhibitory TMS over the right hemisphere resulted in weaker orienting effects in the right (ipsilateral) hemifield compared to the left hemifield. Several studies (referenced above) and theoretical accounts (Duecker and Sack, 2015a, 2015b; Kinsbourne, 1977) suggest that the right hemisphere processes contralateral targets. Its disruption should therefore have an effect on the left hemifield. Several (not mutually exclusive) explanations of the direction of this effect are conceivable, all related to the conceptualization and calculation of spatial orienting effects.

To be specific, subtracting valid from invalid trials combines two distinct cognitive processes into one outcome measure, namely, the initial shift of attention to the cued location and re-orienting if the target appears in the uncued location. In the present study, we decided to follow this conventional analysis approach to allow comparisons with the existing LANT literature. However, it is important to highlight the increased specificity that can be obtained when referencing valid and invalid trials to neutral trials to separate attentional benefits and costs at the behavioral level (Duecker et al., 2013a; Duecker et al., 2017), as well as hemispheric selection and suppression when combined with electrophysiology (Gallotto et al., 2020).

Keeping this in mind, the observed difference between left and right hemifields after network TMS, and within the right hemifield between network TMS and single-node TMS (FEF)/sham, could arise as a consequence of the right hemisphere disruption, in turn leading to a possible disinhibition of the left hemisphere (Kinsbourne, 1977), and eventually resulting in faster re-orienting to the right hemifield (reduced orienting effect). Similarly, attention shifts contingent on leftward cues might be weaker after TMS over the right hemisphere, which may facilitate re-orienting to the right hemifield (again, reduced orienting effect). Besides the possible explanations of what might have caused this result, the stronger effect observed after network TMS remains, and clearly indicates that this method can be promising to obtain more consistent effects compared to single-node TMS.

### (Lack of) effects on alerting and executive control

None of the TMS conditions induced behavioral effects on alerting or executive control. Two previous studies showed TMS effects on alerting and no effects on the executive control after applying rTMS over the right parietal cortex (Xu et al., 2013, 2016). However, as previously mentioned, they presented the targets centrally, supposedly engaging the DAN differently than when targets are lateralized. A more recent study using the LANT reported that transcranial direct current stimulation applied to the DAN nodes affected specifically spatial orienting (Lo et al., 2019) but did not affect other attention components. Similarly to their findings, we also observed TMS effects only on spatial orienting. We previously discussed and outlined the value of null results in NIBS studies (de Graaf and Sack, 2011, 2018). In this case, they were not surprising, and constitute level A null evidence, based on a sophisticated coil positioning strategy, convincing (lack of) effect sizes, and the neural efficacy check provided by the positive effects on spatial orienting (de Graaf and Sack, 2018). Therefore, these null results are meaningful by themselves, and could even be considered to contribute to the function-specificity of our revealed effects on spatial orienting.

### Conclusion

Our results support the hypothesis that network TMS can affect a cognitive function more strongly than modulation of individual nodes within that network. Because many experimental tasks, as well as brain-based disorders, have their basis in networks rather than individual regions, this approach is promising for future experimental and clinical neuromodulation applications.

### Limitations of the study

In the current study, we show that simultaneous inhibition of right FEF and right IPS leads to a stronger reduction of the orienting effect as compared to single-node stimulation. Given that both nodes contribute to the activity of the DAN network, we cannot exclude that sequential stimulation within one node could lead to the same results as stimulation in two nodes. Thus, applying cTBS twice to one node might simply more strongly affect the DAN as compared to single-node stimulation. Owing to the large number of sessions already included in our study, we did not test this hypothesis, but future experiments may include it in their design.

## STAR★Methods

### Key resources table

**Table:** 

REAGENT or RESOURCE	SOURCE	IDENTIFIER
**Software and algorithms**		

Presentation	NeuroBehavioral System	https://www.neurobs.com/
Brain-Voyager	Brain Innovation	https://www.brainvoyager.com/

### Resource availability

#### Lead contact

Further information and request for resources should be directed to and will be fulfilled by the Lead Contact, Stefano Gallotto (stefano.gallotto@maastrichtuniversity.nl).

#### Materials availability

This study did not generate new unique reagents.

### Experimental model and subject details

#### Participants

The study included twenty right-handed participants (5 men, M age: 24.3, range: 19–28). Before each session a written informed consent and a TMS screening form were completed. Only participants with normal or corrected-to-normal vision and without any history of neurological or psychiatric disorders were included. The study was approved by the Ethics Review Committee Psychology and Neuroscience at Maastricht University. Participants received monetary compensation in vouchers (10 €/h) for participation.

### Method details

#### fMRI

To individually localize right FEF and right IPS (regions of interest, ROIs), volunteers participated in an fMRI localizer session in which they performed a simple visual task consisting of two conditions: 1) fixating a black circle presented at the center of a screen, 2) following with their eyes the black circle appearing at one of eight different locations every 500 ms. These eye movements (saccades) activate the nodes of the DAN (Amiez and Petrides, 2009; Corbetta et al., 1998), allowing their identification in each individual participant. fMRI data were acquired at the Maastricht Brain Imaging Center with a Siemens MAGNETOM Prisma 3T scanner (Siemens Medical Systems, Erlangen, Germany). Anatomical data were collected using a 3D ADNI MPRAGE sequence (192 sagittal slices, matrix = 256 × 256, field of view = 256 × 256 mm, slice thickness = 1 mm, no gap, in-plane voxel size = 1 × 1 mm, flip angle = 9°, TR = 2250 ms, TE = 2.6 ms). Functional data were obtained using a T2∗-weighted single shot EPI sequence (32 oblique slices with full-brain coverage, matrix = 64 × 64, field of view = 192 × 192 mm, slice thickness = 3mm, no gap, in-plane voxel size = 3 × 3 mm, flip angle = 90°, TR = 2000 ms, TE = 30 ms). Data were preprocessed and analyzed using the Brain-Voyager QX 2.8 software package with default settings (Brain Innovation, Maastricht, The Netherlands). Intensity inhomogeneity correction and 3D motion correction were performed on the functional data after having aligned the cerebrum to an imaginary plane connecting anterior and posterior commissures (AC-PC space). Next, meshes of both left and right hemispheres were reconstructed, allowing precise TMS coil positioning during the TMS application. Functional and anatomical data were then co-registered and a general linear model was used to identify in the right hemisphere the most significant activation cluster around the intersection of the precentral sulcus and superior frontal sulcus for the localization of the right FEF and along the intraparietal sulcus. In some participants multiple frontal or (more often) parietal clusters were observed. This is not surprising given the different subregions the IPS is composed of (Scolari et al., 2015). If this was the case, the cortex-based alignment approach (Duecker et al., 2013b; Mayrhofer et al., 2019) was used to guide ROIs localization. Functional group data obtained from a cohort of participants who took part in a previous study (Mayrhofer et al., 2019), in which they performed an attention task also known to activate the DAN (i.e. the Posner task, Posner, 1980), were projected on the individual anatomical data of the participants of our study. This was possible because we cortex-based aligned our participants’ anatomy to the group anatomy of that previous cohort. Then, by taking into account anatomical and functional constraints, we chose the individual functional clusters obtained in response to saccadic eye movements that were closest to the functional group data. This procedure allowed us to be consistent in defining functional clusters across our sample. Table S1 reports individual and group coordinates in Talairach space.

#### Neuronavigation

Coil positioning was performed by using the Localite TMS Neuronavigator system version 3.0 (Localite GmbH, St. Augustin, Germany). This frameless system allowed us to precisely target the predefined ROIs at individual level and move the coil from the first to the second site of stimulation in a short period of time (time in between cTBS stimulations across participants: M = 24.4 s, SD = 1.9 s). Since cTBS effects are known to last up to 1 h (Huang et al., 2005), this procedure allowed us to achieve inhibition of both nodes at the same time.

#### TMS

A MagVenture MagPro X100 stimulator equipped with the high-performance cooling system (MagVenture, Farum, Denmark) and a figure-of-eight TMS coil (Cool-B65; inner diameter = 35mm, outer diameter = 75mm) were used to deliver fMRI-guided cTBS and/or sham cTBS over both the right FEF and the right IPS. For real stimulations (the stimulated node in the single-node TMS conditions and both nodes in the network TMS conditions) the coil was manually placed tangentially to the head with the handle/induced current orientation rotated 45° from the midline about an imaginary y axis. For sham stimulations (the non-stimulated node in the single-node TMS conditions and both nodes in the sham condition), the coil was rotated 90° away from the skull about an imaginary Z axis. In this manner participants could feel the coil on the head as well as the sound it generated during stimulation, but no effective stimulation was performed. The protocol consisted of 50-Hz triplets delivered 5 times a second for 40 s (600 biphasic pulses in total). The stimulation intensity (M = 33% of maximum stimulator output, SD = 5.5) was 80% of the individual resting Motor Threshold (rMT), defined as the lowest machine output intensity able to induce a visible twitch in the left index finger for 50% of pulses. The rMT was determined in the first session and used for the other four sessions. The sham condition was obtained by rotating the coil 90° away from the skull, to avoid inducing the magnetic field into the brain. This approach allowed us to mimic the real stimulation, while keeping its fundamental characteristics in terms of TMS protocol and the sound it generates, as well as the position of the coil (Duecker and Sack, 2015a, 2015b).

In a within-subject design, we administered five sequential cTBS stimulations in a counterbalanced order as follows: 1) real FEF → sham IPS, 2) real IPS → sham FEF, 3) sham FEF → sham IPS, 4) real FEF → real IPS, 5) real IPS → real FEF. The latter two conditions both included real cTBS over both DAN nodes, but with either FEF first, or IPS first. We included this condition to evaluate whether the activity of the network was differently affected by the order of stimulation. Although we did not hypothesize specific order effects, they could not be excluded *a priori*. Figure S1 shows a representation of these five conditions and the activation clusters we targeted. The five sessions took place on five different days. Since in each session we applied TMS twice, sessions were separated by at least three days to avoid any carry-over effects. Lastly, to track how participants felt during the stimulation, we collected information on different measures reported in Table S2.

#### Eye tracking

Since the attention task requires participants to covertly focus on one hemifield, an eye tracker Eyelink 1000 system (SR Research, Mississauga, Canada) was used to track gaze position of the right eye. Before starting the task, participants performed a five-point (center, top, bottom, left, and right) calibration and validation procedure. This allowed us to successively track, *post-hoc* sort and remove trials containing eye blinks and/or saccades exceeding 2 degrees of visual angle (M = 3.6% of trials across all conditions, SD = 4.0). The time window of interest started 200 ms before the cue until the appearance of the target.

#### Procedure

At the beginning of each session participants completed a short practice of the task. This assured us that they were able to perform the task properly, namely respond to the appearance of targets accurately and quickly enough while maintaining central fixation. After the practice session the neuronavigation system was prepared for the application of TMS by co-registering the position of participants’ head and the position of the coil. After this procedure we applied cTBS (real and/or sham) twice, once over the right FEF and once over the right IPS (or vice versa) while participants were wearing earplugs to protect them from the sound generated by the coil. Stimulation conditions were counterbalanced across participants. Right after the stimulation participants were seated in front of a computer screen with their head supported by a chin rest at a viewing distance of 75 cm. Once in position, they were asked to start the task, which lasted approximately 40 min. Thus, we assured the effects of TMS lasting up to 1 h (Huang et al., 2005) were effective throughout the task. The procedure was identical for every session.

#### Task and stimuli

Participants performed the Lateralized Attention Network Test (LANT). By comparing reaction times of different cue/target conditions (explained below) this task allows investigating the efficiency of three attention networks: alerting, spatial orienting and executive control (Asanowicz et al., 2012; Callejas et al., 2004, 2005; Chica et al., 2012; Greene et al., 2008). These networks are anatomically separated but highly interacting (Posner and Petersen, 1990).

The task consisted of 720 trials divided in 5 blocks, each composed of 144 trials (plus 4 warm-up trials presented at the beginning of each block). Visual stimuli were presented with the software Presentation (version 19.0, NeuroBehavioral System, Albany, CA) on a gamma corrected 24-inch monitor (Iiyama Pro-Lite B2483HS, Iiyama, Japan) using a 1920 × 1080 (60Hz) mode. Target stimuli consisted of five vertical arrows one on top of another, presented for 200 ms either on the left or right hemifield at 7° eccentricity from the fixation point. When all the arrows were pointing in the same direction (either upward or downward), trials were defined as congruent. When the central arrow was pointing in the opposite direction than the other four arrows, trials were defined as incongruent. Participants were instructed to identify and report whether the central arrow was pointing upward or downward independently of the orientation of the other four arrows, and to respond as quickly and accurately as possible. Targets could be preceded by cues of four types: spatial cues consisted of two double arrowheads next to a central dot either pointing 1) leftward (<<·<<) or 2) rightward (>>·>>), prompting participants’ covert attention toward one hemifield, 3) neutral cues consisted of two double arrowheads pointing in opposite directions (<<·>>), providing temporal but not spatial information and, lastly 4) some trials were not preceded by any cue, thus not providing any spatial nor temporal information. The cue duration was 100 ms, after which there was an interval of 500 ms before the appearance of the target (i.e. stimulus onset asynchrony = 600 ms). Participants responded using a computer keyboard, pressing the up-arrow key (upward orientation) with the right middle finger or the down-arrow key (downward orientation) with the right index finger. In between trials there was a jitter of either 1400, 1600, or 1800 ms. In Figure S2 an example of a trial is shown. The combination of cue type and target hemifield resulted in a design with valid, invalid, neutral, and no cue trials, with a presentation ratio of 4:1:2:2 respectively. Valid trials were more numerous than the other trial conditions to ensure that participants indeed followed the information conveyed by the cue and shifted their attention accordingly.

The three attention network components were obtained by subtracting reaction times of different conditions. The alerting component was isolated by calculating the reaction time difference between neutral and no cue trials. The neutral cue conveys temporal information that allows participants to respond faster compared to trials without any cue. The spatial orienting component was isolated by calculating the reaction time difference between valid (e.g. a right cue with the target appearing on the right hemifield) and invalid (e.g. a right cue with the target appearing on the left hemifield) trials. Participants respond faster to validly cued trials compared to invalidly cued trials. The executive control component was isolated by calculating the reaction time difference between congruent and incongruent trials. Congruent trials are processed faster than incongruent trials since they do not generate cognitive conflict.

### Quantification and statistical analysis

#### Statistical analysis of behavioral data

Statistical analyses were performed on reaction times in trials with correct responses and without eye blinks and/or saccades. This allowed us to only include trials in which participants attended the cued location without moving their eyes, thus assuring that behavioral effects were due to shifts of attention and not eye movements. Furthermore, of the remaining trials outliers at the trial level were removed (>1.5 times the interquartile range away from the 25^th^ or the 75^th^ percentile of the sample), leading to an inclusion in the statistical analyses of 78% of the total amount of trials. To ensure that the LANT attention components we wanted to modulate with TMS were actually present in a normal (sham) condition, individual mean reaction times (RTs) for the three networks were tested with three separate t-tests, confirming alerting, spatial orienting and executive control effects. Once we established that reaction times to targets were indeed modulated as expected, we evaluated effects of TMS on the attention components. Based on previous literature (Greene et al., 2008; Asanowicz et al., 2012), we *a priori* decided to investigate how TMS affected the three attention networks in each hemifield. Three separate 5 × 2 repeated-measures analyses of variance (ANOVAs) were performed, one per each attention component (alerting, spatial orienting, executive control), with TMS condition (FEF, IPS, sham, IPS→FEF, FEF→IPS) and Hemifield (left and right) as within-subject factors. In case of a significant TMS x Hemifield interaction (Bonferroni corrected for multiple comparisons), follow-up t-tests investigated RT differences between left and right hemifields. One subject for the spatial orienting component was excluded from the analysis, as a statistical outlier in the within-subject differences (>1.5 times the interquartile range away from the 25^th^ or the 75^th^ percentile of the sample). Removing this participant also from the analyses on alerting and executive control did not qualitatively change their statistical outcomes.

## Data Availability

• Data reported in this paper will be shared by the lead contact upon request. • This paper does not report original code. • Any additional information required to reanalyze the data reported in this paper is available from the lead contact upon request.
