# Supplementary material for: Concurrent frontal and parietal network TMS for modulating attention
Source: iScience. 2022 Feb 22;25(3):103962. doi: 10.1016/j.isci.2022.103962 (PMC8919227; doi:10.1016/j.isci.2022.103962)
Supplement: Document S1. Figures S1 and S2, Table S1 — and S2 [file mmc1.pdf]

**iScience, Volume *25***

## Supplemental information

# Concurrent frontal and parietal network TMS for modulating attention

**Stefano Gallotto, Teresa Schuhmann, Felix Duecker, Marij Middag-van Spanje, Tom A. de Graaf, and Alexander T. Sack**

**Figure S1. Activation clusters and stimulation conditions, related to STAR Methods**

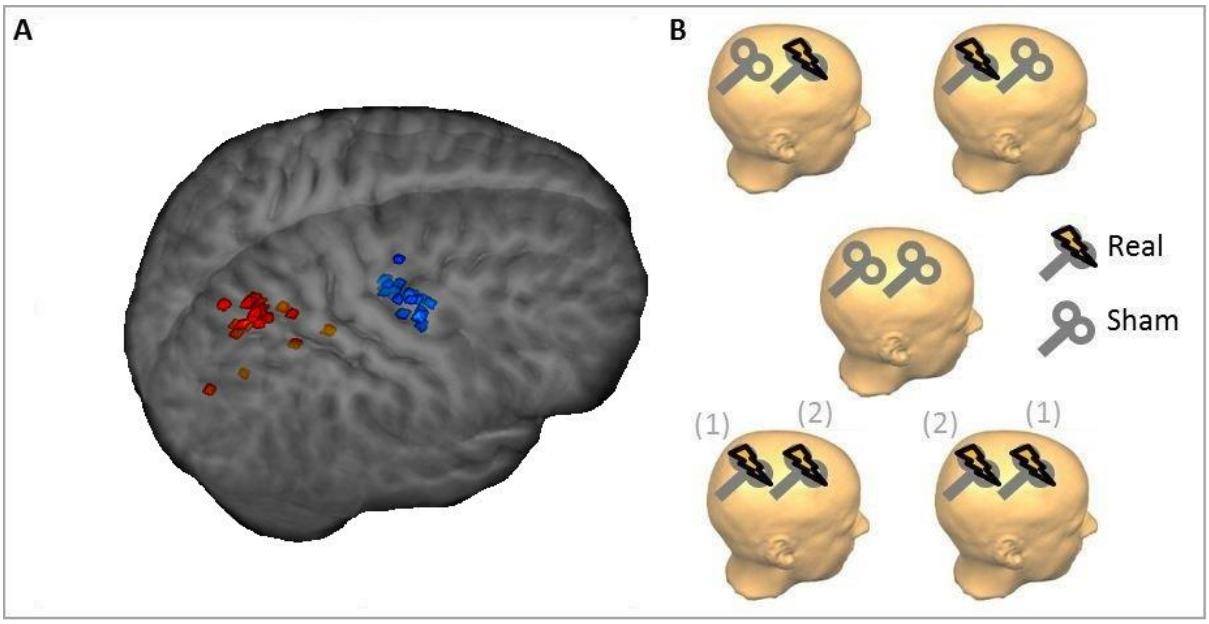


A) FEF and IPS activation clusters (depicted in blue and red respectively) of all participants obtained from saccadic movements and projected on a template brain mesh. B) Stimulation conditions: offline fMRI-guided cTBS was applied over right FEF, right IPS, or both network nodes in both orders of stimulation (IPS→FEF, FEF→IPS); *(1)* = 1st stimulation, *(2)* = 2nd stimulation.

**Figure S2. Lateralized attention network test, related to STAR Methods**

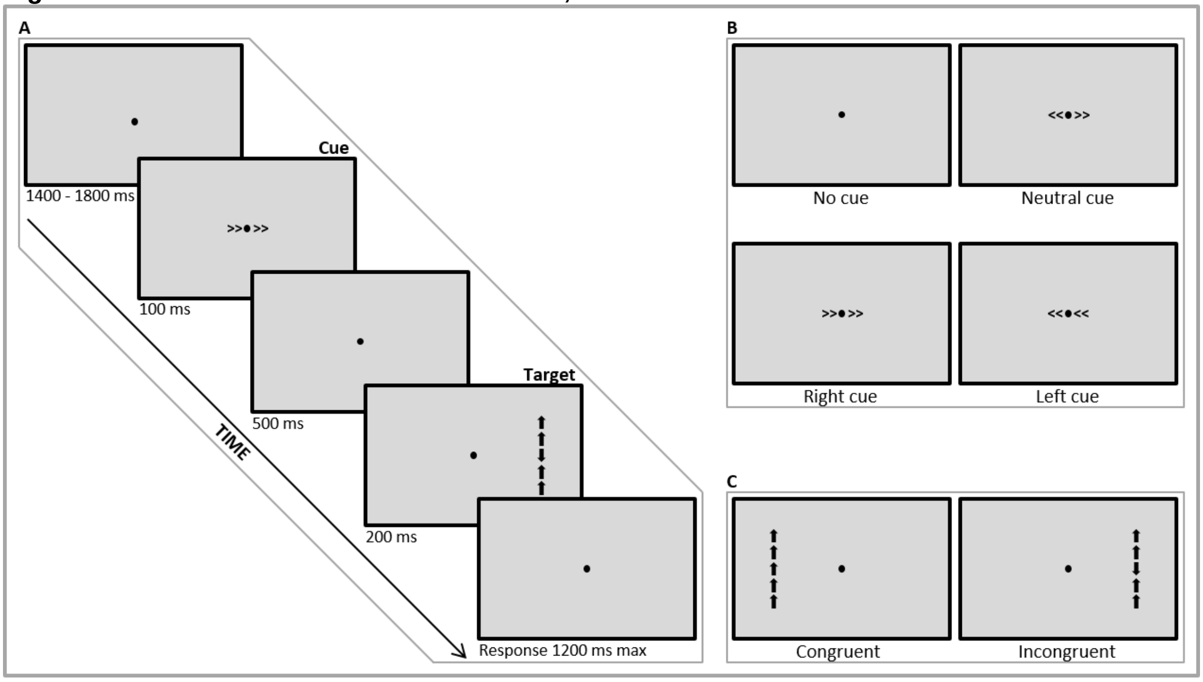


A) Example of a trial. A right cue validly prompts attention toward the right hemifield, where an incongruent target appears. B) Cue conditions (clockwise from top-left: no cue, neutral cue, left cue, and right cue). C) Target types (congruent, incongruent) and possible target locations (left, right).

**Table S1. Individual and group right FEF and right IPS coordinates, related to STAR Methods**

| R FEF | 1 | 2 | 3 | 4 | 5 | 6 | 7 | 8 | 9 | 10 | 11 | 12 | 13 | 14 | 15 | 16 | 17 | 18 | 19 | 20 | M | SEM | Range |
|---|---|---|---|---|---|---|---|---|---|---|---|---|---|---|---|---|---|---|---|---|---|---|---|
| **x** | 26 | 40 | 22 | 29 | 35 | 29 | 28 | 38 | 31 | 37 | 24 | 22 | 36 | 26 | 23 | 40 | 36 | 36 | 38 | 27 | 31 | 1.4 | 18 |
| **y** | -10 | -6 | -4 | -5 | -4 | -12 | -6 | -6 | -2 | -5 | -11 | -12 | -10 | -14 | -13 | -7 | -1 | -11 | -6 | -8 | -8 | 0.9 | 13 |
| **z** | 45 | 46 | 57 | 44 | 48 | 53 | 44 | 52 | 42 | 44 | 40 | 49 | 52 | 46 | 45 | 45 | 46 | 44 | 40 | 51 | 47 | 1.0 | 17 |
| **R IPS** | **1** | **2** | **3** | **4** | **5** | **6** | **7** | **8** | **9** | **10** | **11** | **12** | **13** | **14** | **15** | **16** | **17** | **18** | **19** | **20** | **M** | **SEM** | **Range** |
| **x** | 31 | 20 | 19 | 20 | 30 | 27 | 19 | 22 | 31 | 20 | 21 | 27 | 27 | 29 | 22 | 30 | 18 | 22 | 26 | 30 | 25 | 1.1 | 14 |
| **y** | -35 | -64 | -54 | -62 | -78 | -56 | -59 | -54 | -45 | -56 | -63 | -57 | -60 | -46 | -47 | -57 | -61 | -57 | -53 | -65 | -56 | 2.0 | 42 |
| **z** | 41 | 56 | 49 | 45 | 27 | 55 | 42 | 49 | 54 | 53 | 42 | 53 | 52 | 37 | 47 | 52 | 40 | 55 | 49 | 30 | 46 | 1.8 | 29 |

Talairach coordinates used for the TMS coil positioning of all (20) participants. M = mean, SEM = standard error of the mean.

**Table S2. Subjective experience of TMS, related to STAR Methods**

| | Pleasurable | Neutral | Slightly uncomfortable | Moderately comfortable | Tingling sensation | Muscle twitches | Vision/ perception |
|---|---|---|---|---|---|---|---|
| **SHAM** | - | 18 | - | 1 | 2 | - | 1 |
| **FEF** | - | 12 | 3 | 2 | 5 | 3 | 3 |
| **IPS -> FEF** | - | 13 | 2 | 3 | 3 | 4 | 1 |
| **IPS** | 1 | 11 | 4 | 1 | 4 | 2 | 1 |
| **FEF -> IPS** | - | 12 | 4 | 3 | 8 | 3 | 3 |

Number of participants who experienced the TMS stimulation as pleasurable, neutral, slightly uncomfortable and moderately comfortable e and who experienced tingling sensation, muscle twitches, vision/perception changes during and/or after the stimulation
